# Increased Circulating Osteopontin Levels Promote Primary Tumour Growth, but Do Not Induce Metastasis in Melanoma

**DOI:** 10.3390/biomedicines11041038

**Published:** 2023-03-28

**Authors:** Rafael Saup, Nidhi Nair, Jingyi Shen, Anja Schmaus, Wilko Thiele, Boyan K. Garvalov, Jonathan P. Sleeman

**Affiliations:** 1 European Center for Angioscience (ECAS), Medical Faculty Mannheim, University of Heidelberg, Ludolf-Krehl-Strasse 13-17, 68167 Mannheim, Germany; 2 Mannheim Institute for Innate Immunoscience (MI3), Medical Faculty Mannheim, University of Heidelberg, Ludolf-Krehl-Strasse 13-17, 68167 Mannheim, Germany; 3 Institute of Biological and Chemical Systems—Biological Information Processing (IBCS-BIP), Karlsruhe Institute of Technology Campus North, Building 319, Hermann-von-Helmholtz-Platz 1, 76344 Eggenstein-Leopoldshafen, Germany

**Keywords:** osteopontin, circulating levels, melanoma, tumour growth, metastasis

## Abstract

Osteopontin (OPN) is a phosphoprotein with diverse functions in various physiological and pathological processes. OPN expression is increased in multiple cancers, and OPN within tumour tissue has been shown to promote key stages of cancer development. OPN levels are also elevated in the circulation of cancer patients, which in some cases has been correlated with enhanced metastatic propensity and poor prognosis. However, the precise impact of circulating OPN (cOPN) on tumour growth and progression remains insufficiently understood. To examine the role of cOPN, we used a melanoma model, in which we stably increased the levels of cOPN through adeno-associated virus-mediated transduction. We found that increased cOPN promoted the growth of primary tumours, but did not significantly alter the spontaneous metastasis of melanoma cells to the lymph nodes or lungs, despite an increase in the expression of multiple factors linked to tumour progression. To assess whether cOPN has a role at later stages of metastasis formation, we employed an experimental metastasis model, but again could not detect any increase in pulmonary metastasis in animals with elevated levels of cOPN. These results demonstrate that increased levels of OPN in the circulation play distinct roles during different stages of melanoma progression.

## 1. Introduction

First discovered in 1979 as a transformation specific phosphoprotein [1], osteopontin (OPN) is a multifunctional acidic protein that exists in numerous isoforms [2]. Also called SPP-1 (secreted phosphoprotein 1), BSP-I (Bone Sialoprotein I), nephropontin, 2AR and ETA1 (early T-lymphocyte activation 1), OPN is expressed physiologically and pathologically by various types of cells, reflecting its functional roles in bones, the immune system, and the central nervous system [3,4]. Furthermore, it has been implicated in the pathophysiology of diverse diseases, including cancer, diabetes and autoimmune and neurodegenerative conditions, as well as cardiovascular disease [5,6,7,8,9]. Alternative splicing and alternative translation produce OPN transcripts that encode protein isoforms that can be located intracellularly and extracellularly, while post-translational modification, such as proteolytic processing, phosphorylation and glycosylation, further enriches the complexity of the osteopontin protein isoforms [8,10,11]. Although poorly understood, intracellular OPN is thought to act in the cytoplasm as an adapter protein that regulates various signal transduction pathways, and in the nucleus, where it has been implicated in regulating the mitotic cycle and transcription [12,13,14,15]. However, most research to date has focused on the extracellular functions of secreted OPN, which constitutes an important matricellular protein found in the interstitial and other bodily fluids.

Secreted OPN exerts numerous biological effects through binding to and activating membrane-bound receptors on the surface of target cells. Particular secreted OPN isoforms interact with and signal via a variety of different integrin heterodimer combinations [2,16]. Secreted OPN also binds to specific CD44 isoforms, eliciting a number of cellular responses that are in part integrin dependent [17,18]. Inducible T-cell costimulator ligand (ICOSL) was recently found to act as a further cell surface receptor for OPN [19]. These receptor-mediated events allow secreted OPN to protect cells from apoptosis, and to play physiological roles in bone homeostasis and biomineralization, wound healing, brain development and the regulation of inflammation and immunity [20].

The pathophysiological roles of OPN are also largely mediated by the soluble OPN isoforms and the receptors they activate. This has been particularly intensively studied in the context of cancer, where OPN expression is strongly upregulated in a wide variety of tumours including melanoma, constitutes an important component of the tumour microenvironment, and serves as a prognostically-relevant biomarker for many cancers [21,22,23]. OPN is produced not only by cancer cells in tumours, but also by tumour-associated stromal cells, including cancer-associated fibroblasts (CAFs) and immune cells [24,25,26,27]. Furthermore, senescent tumour and stromal cells produce OPN as part of their senescence associated secretory phenotype, which induces the growth, invasion and migration of non-senescent tumour cells [28,29]. Functionally, OPN expression in the tumour microenvironment is associated with various aspects of progression, including promotion of cell migration, invasion and metastasis, fostering proliferation and tumour growth, enabling tumour cell survival, chemoresistance and stemness properties, induction of epithelial mesenchymal transition, stimulation of angiogenesis and the activation of CAFs, as well as the creation of a tumour-promoting immunosuppressive microenvironment [22,30,31]. In view of this, OPN is receiving increasing interest as a therapeutic target, and a number of approaches are currently in preclinical development, including several antibodies that interfere with the binding of OPN to its receptors, which have shown promise in animal tumour models [7,32,33,34] and other pathological conditions [35,36]. With the recent rapid advances in the use of immune checkpoint inhibitors to treat cancer, the therapeutic targeting of OPN has particularly come to the fore in view of findings that OPN can bypass anti-PD1 immunotherapy [33,37].

The strongly increased expression and secretion of OPN in the tumour microenvironment results in increased levels of circulating OPN (cOPN) in the peripheral blood of patients with various types of cancer, including mesothelioma, glioblastoma, hepatocellular carcinoma, breast, bone and head and neck cancers [38,39,40,41,42,43,44,45], which in some cases has been shown to be prognostically relevant. In cutaneous metastatic melanoma, increased OPN plasma levels have also been detected [46,47]. However, it is currently unclear whether these increased levels of OPN in the blood merely act as surrogate biomarkers of the increased expression of OPN within the tumour microenvironment, or whether they potentially play a functional role in tumour growth and metastasis.

Here we investigated the functional impact of increased circulating levels of OPN on tumour growth and metastasis. To this end we used AAV-mediated transduction in vivo to increase the circulating levels of OPN in healthy immunocompetent mice, before injecting the mice with syngeneic melanoma cells. We found that primary tumour growth was significantly enhanced in response to increased cOPN levels, but this was not associated with a significant increase in lymph node metastasis or distant metastasis to the lungs. These data show that increased levels of OPN in the blood are functionally relevant for tumour progression, with distinct effects on primary tumours and metastases.

## 2. Materials and Methods

### 2.1. Cell Culture

B16F10 mouse melanoma cells were obtained from American Type Culture Collection (ATCC, Manassas, VA, USA) and cultivated in DMEM containing 4.5 g/L glucose (Gibco, Carlsbad, CA, USA) supplemented with 10% fetal bovine serum (FBS; Sigma Aldrich, St. Louis, MO, USA) and 1% penicillin/streptomycin sulfate (Gibco, Carlsbad, CA, USA) at 37 °C in an atmosphere with 5% CO_2_ and 95% humidity. Cells were detached using trypsin-EDTA (0.05%) with phenol red (Gibco, Carlsbad, CA, USA). Cells were regularly tested and confirmed to be mycoplasma-free.

### 2.2. Cloning and AAV Production

Mouse Spp1 (OPN, NM_009263) was cloned into the psubCAG-WPRE-MGH vector [48], which was a gift from Andrey Anisimov and Kari Alitalo. To this end, Spp1 was amplified using the following primer pair with overhangs for NheI (5′-GGGGCTAGCATGAGATTGGCAGTGATTTG-3′; 5′-GGGGCTAGCTTAGTTGACCTCAGAAGATG-3′), purchased from Metabion (Steinkirchen, Germany). The PCR product, as well as the psubCAG-WPRE-MGH vector, were then digested by FastDigest NheI in FastDigest Green Buffer (both Fermentas, Burlington, Canada) at 37 °C for 2 h, followed by inactivation at 65 °C for 5 min. The digested vector was then dephosphorylated at 37 °C for 1.5 h using calf intestinal alkaline phosphatase (CIAP; Thermo Fisher Scientific, Waltham, MA, USA). In a next step, the PCR product and vector were purified by electrophoresis in a 1% gel of LE (low electroendosmosis) agarose (Biozym, Hessisch Oldendorf, Germany) and QIAquick Gel Extraction Kit (Qiagen, Venlo, Netherlands), following the manufacturer’s instructions. Spp1 was then ligated into the psubCAG-WPRE-MGH vector with T4 DNA ligase (Fermentas, Burlington, Canada) at 16 °C overnight and the ligation product was transfected into competent *E. coli*. Colonies that were able to grow on agar plates containing ampicillin were screened by PCR using the following primers 5′-GAGGTCAAAGTCTAGGAG-3′; 5′-AGCGTAAAAGGAGCAACA-3′ (Metabion, Steinkirchen, Germany). The psubCAG-WPRE-OPN vector was then isolated from a validated clone using the QIAGEN Plasmid Maxi Kit (Venlo, Netherlands), following the manufacturer’s instructions and confirmed by sequencing with the following primer pair from Metabion (Steinkirchen, Germany): 5′-CAGTGGATGTTGCCTTTACTTCTAGG-3′; 5′-AGCGTAAAAGGAGCAACA-3′.

Adeno-associated viruses 9 (AAV9) containing psubCAG-WPRE-OPN (OPN-AAV9) or an empty control (S2) plasmid (Control-AAV9) were produced by and purchased from the AAV Gene Transfer and Cell Therapy Core Facility (University of Helsinki, Finland).

### 2.3. AAV Transduction, Blood Collection and OPN ELISA

Animal experiments were performed according to German legal requirements and were approved by the local regulatory authorities (Regierungspräsidium Karlsruhe; approval number AZ35-9185.81/G-260/18). Eight-week-old female C57BL/6J mice were injected intravenously (i.v.) with empty adeno-associated viruses (control-AAV9) or osteopontin expressing viruses (OPN-AAV9) in 100 µL Dulbecco′s Phosphate Buffered Saline (DPBS; Gibco, Carlsbad, CA, USA) at titres of 5 × 10^10^, 1.5 × 10^11^ and 5 × 10^11^ viral particles per mouse (8 mice per group). 100 µL blood was collected from the tail vain, with a syringe containing 8 µL of 15% ethylenediaminetetraacetic acid tripotassium salt dihydrate (K3-EDTA; Sigma-Aldrich, St. Louis, Missouri, USA) in DPBS, 14 days prior to AAV injection, as well as at 7-day intervals following AAV-mediated transduction. At the endpoint, 6 weeks after the AAV injection, blood was collected by cardiac puncture and mixed with 30 µL 15% K3-EDTA. For metastasis assays, blood samples were collected in the same way 14 days before AAV transduction and at the endpoint. Blood samples were centrifugated for 10 min at 4000 rpm and 4 °C. The clear plasma phase was collected, flash frozen in liquid nitrogen and stored at −80 °C. For OPN quantification, plasma samples were diluted 1:500 with Calibrator Diluent RD6-12 and analysed with a Mouse/Rat Osteopontin (OPN) Quantikine ELISA Kit (R&D Systems, Minneapolis, MN, USA), according to the manufacturer’s instructions.

### 2.4. Spontaneous and Experimental Metastasis Models

For the spontaneous metastasis experiments, female C57BL/6JOlaHsd mice were purchased from Envigo (Horst, Netherlands) at the age of 5 weeks and acclimated for 1 week prior to the first blood draw at day −14. Two weeks after the first blood sample was collected (day 1), mice were first injected i.v. with 5 × 10^10^ viral particles in 100 µL DPBS of either OPN-AAV9 or control-AAV9. They were injected 6 days later with 100,000 B16F10 melanoma cells (confirmed shortly before injection to be mycoplasma negative) in 100 µL DPBS subcutaneously (s.c.) into the flank. Tumour size was measured with a caliper. As soon as it reached the maximum permitted diameter of 2 cm (day 28–33), blood, axillary lymph nodes and lungs were collected for analysis. Metastases on the surface of the lungs were counted under a stereo microscope. Tumour and lymph node volumes were calculated using the formula V = L × W × D/2, where L is length, W is width and D is depth.

For the experimental metastasis experiments, 6 to 7-week-old female C57BL/6J mice were equally divided into 2 groups and blood was collected to determine pre-transduction baseline levels of OPN. After 2 weeks, the mice were first injected i.v. with control-AAV9 or OPN-AAV9 at 5 × 10^10^ viral particles per animal in 100 µL DPBS (day 1), followed by i.v. injection of 1 × 10^5^ B16F10 melanoma cells in 100 µL DPBS 6 days later. 28 days after AAV9 injection, blood and lung samples were collected.

Lymph nodes and right superior lung lobes were flash frozen in liquid nitrogen for RNA isolation; the right inferior lobe was frozen in Tissue-Tek OCT; the remaining lung lobes were fixed in 10% formalin and imaged on a Leica MZ10 F stereo microscope to determine the size the metastases, using Fiji/ImageJ [49].

### 2.5. RNA Isolation and Quantitative Real Time PCR (qRT-PCR)

Tissue samples were flash frozen in liquid nitrogen and stored at −80 °C until RNA isolation. For total RNA isolation, the frozen lungs and lymph nodes were transferred into TRIzol reagent (Thermo Fisher Scientific, Waltham, MA, USA), following the manufacturer’s instructions. RNA (3 μg) was treated with RNase-free DNase I, followed by deactivation with EDTA for 10 min at 65 °C. First-strand cDNA was synthesized with random hexamer primers, using dNTP mix and RevertAid H Minus Reverse transcriptase (all from Thermo Fisher Scientific, Waltham, MA, USA). qRT-PCR was performed in a Stratagene Mx3500P qPCR machine (Agilent, Santa Clara, CA, USA) using GoTaq qPCR master mix (Promega, Fitchburg, WI, USA). The relative expression was estimated using the ΔΔCT method, and data were normalized to RPLP0.

The following forward and reverse primers were purchased from Metabion (Steinkirchen, Germany) and used for qRT-PCR: CCL2: 5′-GCTACAAGAGGATCACCAGCAG-3′, 5′-GTCTGGACCCATTCCTTCTTGG-3′; CXCL1: 5′-TCCAGAGCTTGAAGGTGTTGCC-3′, 5′-AACCAAGGGAGCTTCAGGGTCA-3′; CXCL2: 5′-CATCCAGAGCTTGAGTGTGACG-3′, 5′-GGCTTCAGGGTCAAGGCAAACT-3′; IL6: 5′-TACCACTTCACAAGTCGGAGGC-3, 5′-CTGCAAGTGCATCATCGTTGTTC-3′; IL8: 5′-GGTGATATTCGAGACCATTTACTG-3′, 5′-GCCAACAGTAGCCTTCACCCAT-3′; MMP2: 5′-CAAGGATGGACTCCTGGCACAT-3′, 5′-TACTCGCCATCAGCGTTCCCAT-3′; MMP3: 5′-CTCTGGAACCTGAGACATCACC-3′; 5′-AGGAGTCCTGAGAGATTTGCGC-3′; MMP9: 5′-GCTGACTACGATAAGGACGGCA-3′, 5′-TAGTGGTGCAGGCAGAGTAGGA-3′; OPN: 5′-GCTTGGCTTATGGACTGAGGTC-3′, 5′-CCTTAGACTCACCGC TCTTCATG-3′; RPLP0: 5′-GGACCCGAGAAGACCTCCTT-3′, 5′-GCACATCACTCAGAATTTCAATGG-3′; SAA: 5′-GGAGTCTGGGCTGCTGAGAAAA-3′, 5′-TGTCTGTTGGCTTCCTGGTCAG-3′; S100A8: 5′-CCTTGCGATGGTGATAAAAGTG-3′, 5′-CCCAGCCCTAGGCCAGAA-3′; S100A9: 5′-CAAAGGCTGTGGGAAGTAATTAAGA-3′, 5′-AGCCATTCCCTTTAGACTTGGT-3′; TGFβ1: 5′-AAGTTGGCATGGTAGCCCTT-3′, 5′-GCCCTGGATACCAACTATTGC-3′; TNFα: 5′-GCCTCTTCTCATTCCTGCTTG-3′, 5′-CTGATGAGAGGGAGGCCATT-3′; Trp1: 5′-GCTGGAGAGAGACATGCAGGA-3′, 5′-AGTGCAGACATCGCAGACGTT-3′. 

### 2.6. Statistical Analysis

All data are presented as mean ± SEM; “n” indicates the number of independent biological replicates (separate animals). For pairwise comparisons, statistical analysis was performed using the Mann Whitney test for quantification of lymph node and lung metastases and unpaired, two-tailed Student’s *t*-test for the remaining experiments, as indicated in the corresponding figure legends. For time course experiments, repeated measures two-way ANOVA with Šídák’s multiple comparisons tests was used. A *p*-value <0.05 was set as a threshold for statistical significance: * *p* < 0.05, ** *p* < 0.01, *** *p* < 0.001, **** *p* < 0.0001; ns *p* ≥ 0.05 (not significant).

## 3. Results

### 3.1. Adeno-Associated Virus-Mediated Transduction Leads to a Sustained Increase in Circulating Osteopontin Levels without Affecting OPN Expression at Potential Metastatic Sites

**Figure 1:**
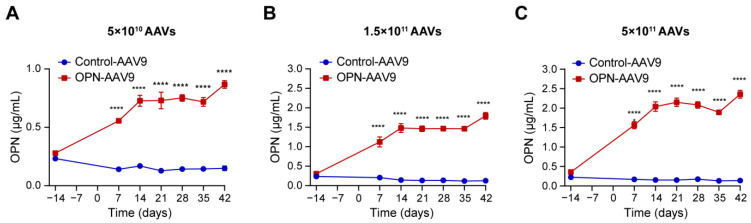
Transduction with OPN-expressing AAV9 increases cOPN levels. (**A**–**C**) C57BL/6 mice were injected i.v. with empty adeno-associated viral vector (Control-AAV9) or osteopontin-expressing adenoviral vectors (OPN-AAV9) at titres of 5 × 10^10^ (**A**), 1.5 × 10^11^ (**B**) and 5 × 10^11^ (**C**) viral particles per mouse at day 0. Blood was collected from the animals 14 days before virus injection to determine pre-transduction levels, as well as at 7-day intervals following AAV-mediated transduction. OPN levels in the plasma were determined by ELISA. Groups were compared using repeated measures two-way ANOVA with Šídák’s multiple comparisons tests; n = 4 for all groups. **** *p* < 0.0001.

In order to achieve a sustained increase in circulating OPN (cOPN) expression, we used an adeno-associated virus (AAV)-based transduction approach. AAV9 viruses expressing either OPN or empty vector were injected intravenously into mice at different titres and the levels of OPN in the plasma were monitored at regular intervals after transduction. All tested titres led to a significant increase in the circulating levels of OPN, already one week after transduction with OPN-AAV9, compared to mice injected with control viruses. At 2 weeks, the levels reached a plateau, with only moderate further increase until day 42 (Figure 1A–C).

**Figure 2:**
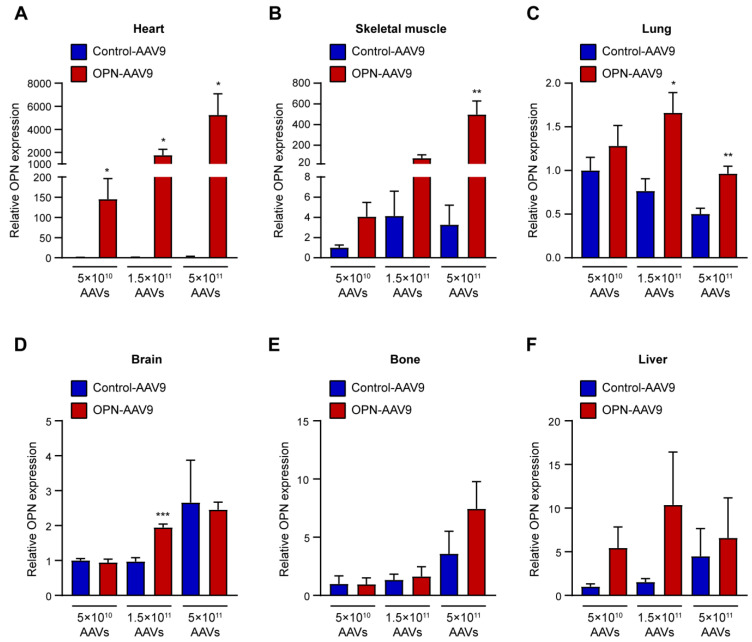
OPN expression in different tissues of transduced animals. (**A**–**F**) C57BL/6 mice were injected i.v. with empty adeno-associated viral vectors (Control-AAV9) or osteopontin expressing adenoviral vectors (OPN-AAV9) at titres of 5 × 10^10^, 1.5 × 10^11^ and 5 × 10^11^ viral particles per mouse. At the end point of the experiment, RNA was isolated from various organs, including skeletal muscle (**A**), heart (**B**), lung (**C**), brain (**D**), bone (**E**) and liver (**F**), and the expression levels of osteopontin were assessed using qPCR. For each titre the control and OPN groups were compared using Student’s *t*-test; n = 4 for all groups. * *p* < 0.05, ** *p* < 0.01, *** *p* < 0.001.

AAV9 vectors have been described as having a relatively broad tropism to various organs of the body including the lung, liver, skeletal muscle, heart and central nervous system [50]. To assess which organs had been targeted by OPN-AAV9 in our system, we determined OPN levels in different organs following viral transduction. The strongest and most robust upregulation of OPN levels at all titres was achieved in the heart (Figure 2A). None of the other organs that were examined showed a significant increase in OPN at the lowest titre of 5 × 10^10^ viral particles per animal (Figure 2B–F). At higher titres, a significant increase was also observed in other organs including skeletal muscle (Figure 2B), lung (Figure 2C) and brain (Figure 2D).

Based on these results, we selected the lowest titre for further experiments since it led to a robust upregulation of cOPN, but without impacting the local expression of OPN in common metastatic sites such as the lung, brain, liver or bones.

### 3.2. Increased cOPN Upregulates Various Factors Related to Tumour Progression

**Figure 3:**
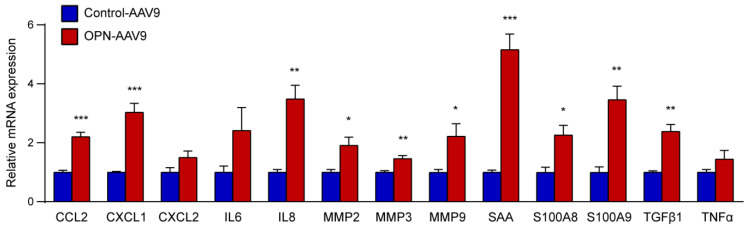
Circulating OPN increase enhances the expression of multiple factors linked to tumour development. RNA was isolated from the lungs of mice transduced with 5 × 10^10^ control or OPN-expressing AAVs. The expression of the indicated transcripts was assessed by qPCR. For each transcript, the control and OPN groups were compared using Student’s *t*-test; n = 4 for all groups. * *p* < 0.05, ** *p* < 0.01, *** *p* < 0.001.

Next, we examined whether elevated circulating levels of OPN can affect the expression of various factors that have been linked to different aspects of tumour progression, and which have been shown to be regulated by OPN [51,52,53,54,55,56]. Specifically, we screened a panel including the chemokines CCL2, CXCL1 and CXCL2, interleukins (IL) 6 and 8, matrix metalloproteinases (MMP) 2, 3 and 9, serum amyloid A (SAA), S100 family members, including S100A8 and S100A9, transforming growth factor β1 (TGFβ1), and tumour necrosis factor α (TNFα). Intriguingly, the majority of the examined factors showed significantly elevated levels in the lungs of mice transduced with OPN-AAV9 compared to control animals (Figure 3).

This indicates that increased cOPN can promote the expression of various factors linked to the growth and dissemination of tumours, prompting us to assess whether it may have an impact on cancer growth and metastasis.

### 3.3. Circulating OPN Increase Promotes Primary Tumour Growth in a Mouse Model of Melanoma

**Figure 4:**
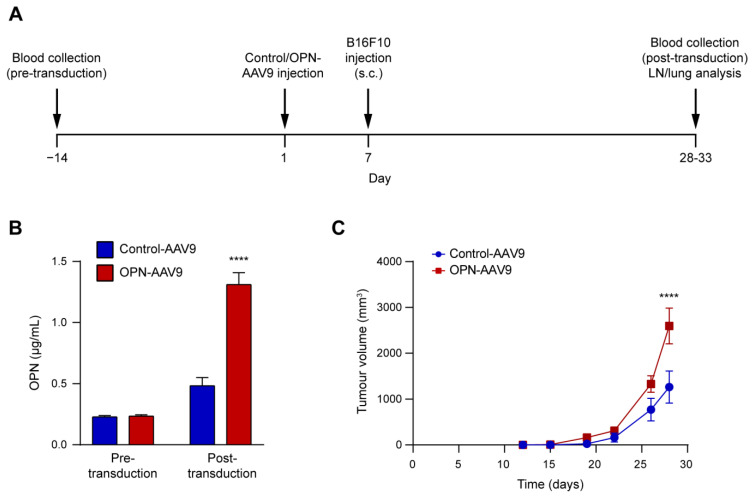
Increased cOPN levels promote melanoma growth. (**A**) Schematic illustration of the experimental time course. Mice were first injected i.v. with control-AAV9 and OPN-AAV9 at 5 × 10^10^ viral particles per animal, and 6 days later they were transplanted with 1 × 10^5^ B16F10 melanoma cells s.c. Tumour growth was monitored, and at the end of the experiment blood, lymph nodes and lungs were collected for analysis. Additionally, blood was collected 14 days before viral transduction to determine pre-transduction baseline levels of OPN. (**B**) Plasma OPN levels pre- and post-transduction were determined by ELISA. Control and OPN-AAV9 transduced animals were compared using Student’s *t*-test. (**C**) Tumour size following s.c. transplantation of B16F10 melanoma cells. Groups were compared using repeated measures two-way ANOVA with Šídák’s multiple comparisons tests; n = 6 for Control-AAV9 and n = 7 for OPN-AAV9. **** *p* < 0.0001.

To determine whether an increase in cOPN levels affects the growth of primary tumours, we used a mouse model of melanoma. C57BL/6 mice were first injected intravenously with control and OPN-expressing AAV9 at 5 × 10^10^ viral particles per animal. Six days later, syngeneic B16F10 melanoma cells were transplanted subcutaneously into the mice (Figure 4A). Analysis of OPN levels in the plasma indicated that the OPN-transduction significantly increased cOPN compared to control animals (Figure 4B). Importantly, the mice with increased cOPN levels showed enhanced B16F10 tumour growth (Figure 4C).

### 3.4. Spontaneous Melanoma Metastasis Is Not Altered by Increased cOPN

**Figure 5:**
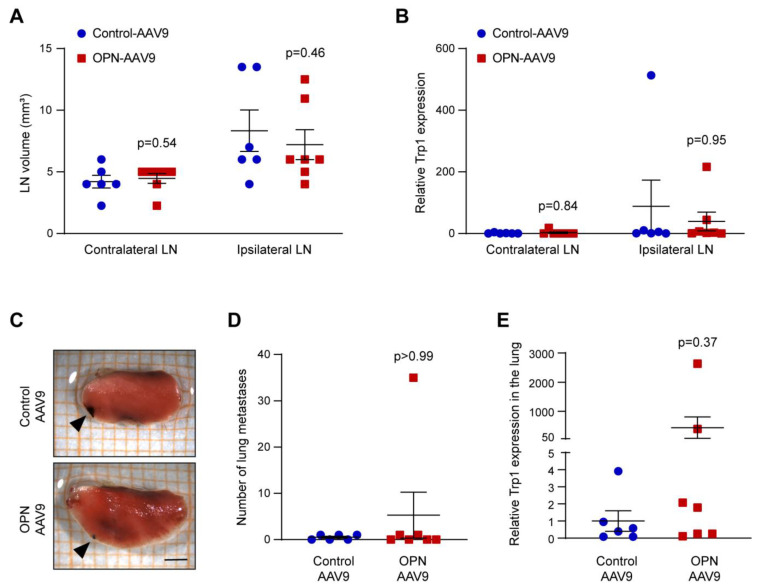
An increase in cOPN does not enhance melanoma lymph node and lung metastasis. Mice were transduced with control-AAV9 and OPN-AAV9 at 5 × 10^10^ viral particles, then injected with B16F10 melanoma cells s.c. At the end point, axillary lymph nodes were analysed. (**A**) Volume of contralateral and ipsilateral axillary lymph nodes (LN). (**B**) Expression of the melanoma marker Trp1 in contralateral and ipsilateral axillary lymph nodes measured by qPCR. (**C**) Representative images of B16F10 metastatic nodules in the lungs (indicated by black arrowheads). Scale bar: 2 mm. (**D**) Number of metastatic nodules in the lungs. (**E**) Expression of the melanoma marker Trp1 in the lungs. Control and OPN groups were compared using the Mann Whitney test; n = 6 for Control-AAV9 and n = 7 for OPN-AAV9.

We next examined whether the increased circulating levels of OPN can influence the ability of melanoma cells to metastasise from primary tumours. Metastasis of cancer cells to regional lymph nodes is often an early event in cancer dissemination, which may represent an important route for further spread to distant organs and generally strongly correlates with the ability to form distant metastases [57]. We therefore next analysed the lymph nodes of mice with elevated OPN levels and controls. We could not detect any significant differences in the volume of the lymph nodes that were either ipsilateral or contralateral to the tumour (Figure 5A). We have recently established a sensitive method for the quantitative detection of disseminated melanoma cells, based on the expression of the melanocytic marker Trp1, which is particularly useful in cases with low metastatic burden [58]. Quantification of Trp1 expression levels by qPCR, however, also did not reveal any difference between control and OPN-AAV9 transduced animals (Figure 5B).

We next asked if the increased levels of cOPN can enhance the propensity of melanoma cells to metastasise to distant organs. Quantification of metastatic nodules in the lungs did not show a significant difference between the control and OPN-AAV9 transduced mice (Figure 5C,D). Since the metastatic burden was relatively low, we additionally carried out qPCR for Trp1, as above, but this analysis also did not detect a significant increase in the OPN-AAV9 group (Figure 5E).

### 3.5. Increased cOPN Does Not Enhance Organ Colonisation and Metastasis Formation in an Experimental Metastasis Model

**Figure 6:**
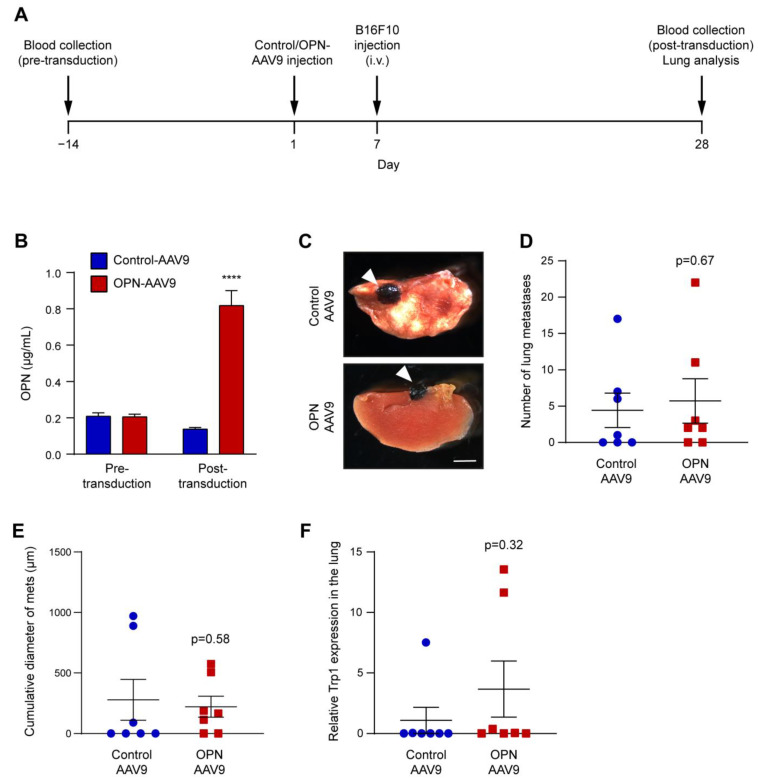
Increased cOPN does not promote lung metastasis in a melanoma model of experimental metastasis. (**A**) Schematic illustration of the experimental time course. Mice were first injected i.v. with control-AAV9 and OPN-AAV9 at 5 × 10^10^ viral particles per animal, and 6 days later transplanted with 1 × 10^5^ B16F10 melanoma cells i.v. Blood and lungs were collected for analysis 28 days after the injection of the AAVs. Additionally, blood was collected 14 days before viral transduction to determine pre-transduction baseline levels of OPN. (**B**) Plasma OPN levels were determined by ELISA. Control and OPN-AAV9 transduced animals were compared using Student’s *t*-test; n = 7 for Control-AAV9 and n = 6 for OPN-AAV9. (**C**) Representative images of B16F10 metastatic nodules in the lungs (indicated by white arrowheads). Scale bar: 2 mm. (**D**) Number of metastatic nodules in the lungs. (**E**) Quantification of the metastasis size (cumulative diameter of all measured metastases). (**F**) Expression of the melanoma marker Trp1 in the lungs. Control and OPN groups in (**D**–**F**) were compared using the Mann Whitney test; n = 7 per group. **** *p* < 0.0001.

While the spontaneous metastasis model did not show a difference between mice with elevated cOPN and controls, it remained conceivable that OPN may impact later stages of the metastatic process, which cannot be clearly revealed in this model. To test whether this may be the case, we employed a second model of metastasis with direct injection of tumour cells in the circulation through the tail vein, which targets them to the lungs. This model is well suited for examining later stages of metastasis, including extravasation, organ colonisation and subsequent cancer growth. In this model, injection of control-AAV9 and OPN-AAV9 was followed 6 days later by injection of B16F10 melanoma cells in the tail vein. After 28 days, lungs were dissected and analysed (Figure 6A). As in the spontaneous metastasis model, OPN-AAV9 transduction led to a significant increase in plasma OPN levels (Figure 6B).

We did not observe a difference in the number of pulmonary metastases between control animals and those with elevated cOPN (Figure 6C,D). In addition, the size of pulmonary metastasis was the same in both groups (Figure 6E). Finally, assessment of Trp1 expression levels also did not reveal a significant difference between the metastatic burden of the control- and OPN-AAV9 transduced animals (Figure 6F).

## 4. Discussion

A growing body of evidence highlights the crucial and pleiotropic roles of OPN in cancer development. An involvement of OPN has been suggested at every step of tumour progression and across a very wide range of tumour types. The vast majority of the studies have focused on OPN produced locally within the tumour—either by tumour cells or by stromal cells within the tumour microenvironment. However, increased levels of OPN are observed not only inside tumours but also in the circulation of tumour patients. It remains poorly understood, though, whether such a systemic OPN increase can have additional effects on tumour progression, on top of those elicited by OPN produced within the tumour tissue. Our study set out to investigate this question.

Recent work has shown that cOPN plays crucial functions in normal physiology, e.g., during maintenance of bone homeostasis ([59], as well as in pathological conditions, such as respiratory failure [60] and inflammatory lung disease [61]. While the levels of cOPN are also increased in multiple cancers [7], the functional consequences of this increase have remained insufficiently understood. To examine the effects of increased cOPN on tumour progression we selected a model of melanoma. Melanoma is the most malignant skin cancer, with a rapidly rising incidence worldwide [62]. A combination of targeted therapy and immune checkpoint inhibitors have considerably improved the treatment of melanoma patients in recent years [62,63]. Nevertheless, most patients with metastatic melanoma have either limited response rates to such therapies or eventually develop resistance against them, emphasising the need to explore novel therapeutic targets for this disease. Osteopontin has attracted considerable attention in this context. Several studies have reported increased levels of OPN in melanoma, similarly to other tumours [7]. This has been shown directly in tumour tissue compared to benign lesions in a number of previous reports [28,47,64,65,66,67]. In addition, transcriptomic analyses have revealed that OPN is one of the most highly upregulated proteins in metastatic melanoma compared to benign nevi [67]. Furthermore, the levels of OPN are also elevated in the blood of melanoma patients compared to healthy controls [46,47,68].

Studies aimed at determining whether OPN expression is associated with metastasis in cutaneous melanoma patients, on the other hand, have produced considerably more divergent findings. Some papers based on transcriptomic analyses or qPCR have reported increased OPN mRNA levels in patients with metastatic melanoma compared to primary (non-metastatic) melanomas [64,65]. A higher level of OPN protein was found in distant melanoma metastases compared to primary tumours in some studies [28], but not in others [69], and one report even found decreased OPN levels in melanoma metastases relative to primary tumours [47]. Several studies that assessed OPN protein levels by immunohistochemistry in primary vs. metastatic cutaneous melanomas did not reveal any difference [65,67,70]. One report found a correlation between high OPN expression in tumours and increased risk of lymph node metastasis, as well as a marginal decrease in survival, which is closely linked to distant metastasis formation [71]. By contrast, a separate study found no correlation between OPN expression and overall or recurrence-free survival in melanoma patients [68].

Analyses of a potential link between cOPN levels in plasma and melanoma metastasis have also not provided a clear-cut picture. Filia et al. detected significantly higher OPN plasma levels in untreated stage IV (metastatic) melanoma patients compared to stage I-III patients, but found no significant correlation between OPN levels and patient survival [46]. Maier et al. similarly reported increased OPN levels in metastatic melanomas compared to non-metastatic ones, while at the same time finding no correlation with lymph node metastasis and a reduction of OPN protein levels in metastases compared to primary tumours [47]. In summary, while OPN is clearly increased in melanoma patients relative to healthy controls, it remains currently uncertain whether either local OPN expression in the tumour or OPN levels in the circulation correlate with melanoma metastasis and disease prognosis in human patients.

A previous publication has provided intriguing indications that cOPN could promote the growth of distant tumours in an animal model [72]. In this study, the authors showed that breast carcinomas could instigate the growth of normally indolent tumour cells transplanted at distant locations. Furthermore, when weakly metastatic cancer cells were intravenously transplanted, the formation of metastatic foci in the lungs was increased in mice carrying instigating tumours compared to controls. This was accompanied by the recruitment of bone marrow derived cells in the stroma of the instigated tumours. Importantly, OPN levels were increased in the plasma of mice bearing instigating tumours and silencing of OPN in the instigating cancer cells abrogated the enhanced growth of distant tumours and metastases, as well as bone marrow-derived cell recruitment to them [72]. This suggests that increased cOPN could directly promote metastatic growth at the distant sites, but it is also possible that OPN expression by the tumour cells may have secondary effects on other molecular or cellular components within the instigating tumours, which eventually promote the growth of the distant tumours.

In the present study we decided to use an adeno-associated virus (AAV)-based delivery method, in order to increase cOPN already before tumour initiation. Adeno-associated viruses are considered particularly suitable vectors for the delivery of exogenous genes in animals, as they are non-pathogenic and elicit no or only very mild immune responses [73]. The AAV9 serotype that we employed can potentially target various tissues. However, when using a moderate titre, local levels of expression were only upregulated in the heart, in line with the reported cardiotropism of this vector [74]. At the titre used, the expression levels of OPN were not locally upregulated in the organs that can represent potential targets of metastasis in our model, especially the lung. This was important, as we wanted to focus on the effects of cOPN that would more closely mimic the situation observed in tumour patients, as opposed to local effects at premetastatic sites due to ectopic OPN expression in the target tissue. Thus, the heart represents a suitable source of OPN production, as cardiac metastases are very rare [75]. Despite the limited tissue distribution of OPN transduction, we could achieve stable and significant increase of cOPN levels. In this context, the choice of the B16F10 melanoma cell model was also pertinent, since these cells produce relatively low levels of osteopontin [76,77], allowing us to achieve an increase in cOPN considerably above that elicited by the presence of the primary tumour alone.

Using the above approach, we observed a significant acceleration of primary melanoma growth. This is in line with a considerable body of evidence that has reported tumour promoting functions of OPN in melanoma [23]. In B16 cells, for example, OPN enhances proliferation through the ERK/MAPK signalling pathway [76]. Using OPN knockout mice, Kumar et al. demonstrated that stromal osteopontin enhances the growth and angiogenesis of B16 melanoma tumours, as well as their cancer stem cell-like properties, in an ERK2-dependent manner [78]. In addition to changes in the properties of cancer cells, OPN can also modulate cells in the tumour microenvironment to promote primary tumour growth [22]. For instance, OPN activated macrophages in a B16 melanoma model, which led to a prostaglandin E2 (PGE2)-dependent increase in angiogenesis and tumour growth [77]. In addition, it was shown that OPN can potently suppress cytotoxic T cell proliferation and activation, promoting tumour immune evasion [37]. Furthermore, OPN reprograms fibroblasts into a cancer-associated fibroblast (CAF)-like proinflammatory state, which is linked to enhanced tumour growth [79].

In contrast to the effect of cOPN on primary tumour growth, we could not detect a significant change in melanoma metastases either to the lymph nodes or to the lung. This may appear unexpected, considering that a number of studies have demonstrated prometastatic functions of OPN in melanoma [76,78,80], as well as other tumours [7]. However, the existing literature has focused on local effects within the primary tumour, where OPN produced by tumour or stromal cells can promote various functions linked to metastatic dissemination, including migration, invasion and epithelial-mesenchymal transition [7,76,78,80]. Evidence whether systemic elevation of OPN in the circulation can have direct effects on metastasis, however, has so far been lacking and our data demonstrate that at least in the model that we employed, it does not play a significant role.

The absence of an effect on metastasis in the spontaneous metastasis model might be due to the lack of sufficient time for metastasis formation before the animals had to be killed due to the primary tumour reaching a critical size. Moreover, it is perfectly possible that cOPN primarily acts by creating a (pre)metastatic niche at distant sites such as the lung, which may not be enough to induce metastatic growth, if a sufficient number of cancer cells cannot disseminate away from the primary tumour within the time-frame of the experiment. To address these potential limitations of the spontaneous metastasis model, we employed a second, experimental metastasis model with direct tumour cell injection into the tail vein. In this model cancer cells are rapidly targeted to the lungs, circumventing the early steps of the metastatic cascade, which should allow any effects of a premetastatic niche on cancer cell seeding and organ colonisation to become apparent. However, analysis of metastatic burden in this model also did not reveal a significant increase in the animals with elevated cOPN.

OPN exists in a variety of isoforms, which may differ in their physiological properties. The murine OPN isoform employed in our experiments has been commonly used in the literature [15,81,82] and was shown to be more potent than other OPN isoforms in promoting pulmonary metastasis formation when overexpressed in B16F10 melanoma cells [81]. Nevertheless, it remains conceivable that it might have limited activity in our AAV9-mediated, systemic transduction-based system. However, we did observe a clear effect of increased cOPN on primary tumour growth in our experiments. Furthermore, we showed that a number of factors which can be regulated by OPN were markedly increased in animals with elevated levels of cOPN. These findings demonstrate that the cOPN used in our system was physiologically active and capable of stimulating various downstream effects linked to tumour progression. We therefore conclude that cOPN stimulates primary melanoma growth, but not the efficiency of metastatic seeding, nor the colonisation of secondary sites. Despite the fact that cOPN induced the expression of tumour progression-relevant genes in the lung, it was not sufficient to promote metastasis initiation or growth in the pulmonary environment, indicating that cOPN has context-dependent effects on the growth of primary and secondary tumours. These findings contribute to the understanding of the multifaceted functions of OPN during tumour progression and have implications for potential OPN-targeting therapeutic approaches in melanoma.

## Data Availability

The data presented in this study is contained within the article or are available on request from the corresponding authors.
